# Nontargeted homologue series extraction from hyphenated high resolution mass spectrometry data

**DOI:** 10.1186/s13321-017-0197-z

**Published:** 2017-02-23

**Authors:** Martin Loos, Heinz Singer

**Affiliations:** 1 Swiss Federal Institute for Aquatic Science and Technology (Eawag), 8600 Dübendorf, Switzerland; 2 Institute of Biogeochemistry and Pollutant Dynamics, ETH Zürich, Zurich, 8092 Switzerland

**Keywords:** Homologue series, Mass spectrometry, Liquid chromatography, Nontarget screening, Sewage effluent, Surfactants

## Abstract

**Background:**

A large proportion of polar anthropogenic compounds routinely released into the environment comprises homologue series, i.e., sets of chemicals differing in a repeating chemical unit. Using analytical techniques such as liquid chromatography coupled to high-resolution mass spectrometry (LC-HRMS), these compounds are readily measurable as signal sets with characteristic differences in mass and typically retention time. However, and despite such distinct characteristics, no computational approach for the direct, simultaneous and untargeted detection of all such signal sets comprising both LC and HRMS information has to date been presented.

**Results:**

A fast two-staged approach has been developed to extract LC-HRMS signal patterns which can be indicative of homologous analytes. In a first stage, a *k*-d tree representation of picked LC-HRMS peaks is used to extract all feasible 3-tuples of peaks with restrictions in, e.g., mass defect differences. A second stage then recombines these 3-tuples to larger series tuples while ensuring smooth changes in their retention time characteristics. This unsupervised approach was evaluated for ten effluent samples from Swiss sewage treatment plants (STPs), in both positive and negative electrospray-ionization.

**Conclusions:**

Beside recovering all continuous series of previously identified homologues, substantial fractions of nontargeted peaks could subsequently be assigned into very diverse peak series, although assignments were often not unique. The latter ambiguities were resolved by a self-organizing map technique and revealed both distinctive series meshing and rivaling combinatorial solutions in the presence of isobaric or gapped series peaks. When comparing STPs, several ubiquitous yet partially low-frequent series mass differences emerged and may prioritize future identification efforts. The presented algorithm is freely available as part of the R package *nontarget* and as a user-friendly web-interface at www.envihomolog.eawag.ch.

**Graphical Abstract:**
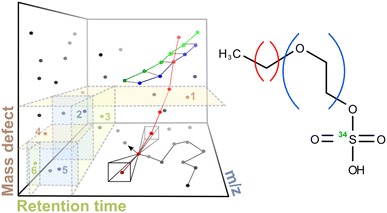
Search for systematic series indicative of homologous compounds is based on a partitioned representation of LC-HRMS signal characteristics. This nontargeted search first extracts series triplets in a nearest-neighbour walk and then recombines them to larger ones. For illustration, the two dimensions involving mass defect characteristics are represented by one only

**Electronic supplementary material:**

The online version of this article (doi:10.1186/s13321-017-0197-z) contains supplementary material, which is available to authorized users.

## Background

Homologue compounds differing in a common chemical subunit are regularly addressed in different areas of research. They have been focused on in fields as diverse as toxicology [1–3], biopolymers [4–7] food control [8, 9] and oil processing [10, 11]. In environmental research, natural and anthropogenic homologue sources have been detected in various media, with Surface Active Agents (Surfactants) even classified as High Production Volume Chemicals (HPVC) [12–19]. Not surprisingly, the analytical detection of homologue series (HS) has therefore been of great interest. Among the methods used, liquid chromatography (LC) and high-resolution mass spectrometry (HRMS) have found abundant application to detect polar and semi-polar HS with both high sensitivity and specificity [20]. However, while most applications have targeted a priori known or suspected HS, rather few nontargeted approaches have been established to extract the LC-HRMS signals of yet unknown HS [21]. Concerning the latter, and as compared to non-homologous compounds, the regular patterns in LC-HRMS signals caused by the repetitive HS chemical units enable a specific fingerprinting. LC-HRMS has therefore potential to routinely single out yet unknown signal series of, e.g., emerging contaminants, yet unidentified transformation products or differently ionized species of the same HS which otherwise evade targeted approaches. Once listed, the repetitive signals of individual or grouped HS would allow for averaged masses and additional peak relations to improve deisotoping, blank removal and finally their identification via complementary analytical methods (reference standards, MS^n^) [22, 23].

Using mass spectrometric information, Kendrick mass defect plots and their extension to more than one type of chemical HS unit have been one popular method to determine the presence of signal patterns caused by unknown HS [11, 22, 24]. Another methodological branch has relied on extensive molecular formula fitting to detect regular patterns among measured classes of compounds, visualized by, e.g., van Krevelen diagrams or carbon versus mass plots [25–27]. Yet others have proposed a projection on regularly spaced vectors for HS pattern recognition [28]. Main drawbacks with these first approaches arise, inter alia, from either the restriction to a fixed set of basic HS units or the requirement to derive unique molecular formulas for demanding numbers of measured masses. Any available information from the orthogonal chromatographic dimension is therein omitted—in spite of the often systematic differences in retention time (*RT*) among the homologues of a series [29, 30]. Methods to embrace chromatographic information and to combine it with HRMS data for signal series detection are however scarce. For instance, Pietrogrande and coworkers have proposed autocovariance functions to reveal joint regularities in mass and *RT* differences [31–34]. Here, one major LC-related drawback is that *RT* differences cannot be easily linearized to align with autocorrelated differences in homologue masses because *RT* differences in a series are often not constant, vary significantly between different HS found in the same sample and can hardly be predicted in nontargeted analysis. Second, retracting and localizing single HS from autocovariance functions may not be straightforward. Third, infrequent HS may simply be masked by noise or the autocorrelation of more frequent HS. In contrast, other methods embracing both LC and HRMS information have rather aimed to aggregate data for comparison of samples, and do not aim at a detection of individual HS [4].

From a data mining perspective, the unsupervised extraction of regular HS patterns is indeed intricate, even from a list of picked signal peaks. As noted elsewhere [35], an exhaustive pairwise peak comparison to find regular mass differences is a time-consuming task, not to speak of computing all possible series of such mass differences. Fortunately, differences in HS mass and *RT* can be restricted and their search optimized through appropriate metric data structures. To this end, a fast two-staged computational strategy to extract systematically spaced peak series from electrospray-ionization (ESI) LC-HRMS measurements is presented. The novel approach detects signal series even when (a) HS are not dominating a complex sample matrix, (b) no deisotoping or blank-subtraction was run beforehand, (c) signal peaks with differing measurement uncertainties exist in the same sample, (d) only limited prior HS information is available, (e) different HS units occur and (f) combinatorial ambiguities arise. The approach is evaluated for ten sewage treatment plant (STPs) effluent samples, both for revealing common patterns and for recovering the series of a priori identified HS compounds.

## Methods

Based on a definition of LC-HRMS signal series which can be caused by homologous compounds, series detection progresses in two stages. A first stage extracts the set *S*_3_ of feasible 3-tupels (triplets) of peaks, while a second stage recombines them to larger tuples of *n* > 3 in a stepwise manner.

### Series definition

A series *k* of length *n* ≥ *n*_*min*_ is defined as the tuple *S*_*n,k*_ = (*p*_1*,k,*_ *…, p*_*n,k*_) of picked LC-HRMS signal peaks *p* = {*m/z, RT, intensity*}, ordered by increasing *m/z* of the series peaks. *S*_*n*_ denotes the set of all such series tuples having length *n*. Peaks being adjacent in a tuple are assumed to only differ in a repetitive and possibly unknown chemical unit or functional group, e.g., CH_2_ or OH. As a result, changes in the mass differences Δ*m/z* between any two adjacent series peaks *p*_*j,k*_ and *p*_*j*+1*,k*_ must remain within an error margin of [−4*ε; *4*ε*]. *ε* here denotes a maximum ± *m/z* measurement error and may depend on *m/z* or peak intensity [36]. The Δ*m/z* of all series in a LC-HRMS data set range within lower and upper bounds Δ*m/z*_*min*_ and Δ*m/z*_*max*_, a priori set as the considered mass range of chemical units at given charges *z*.

Furthermore, Δ*m/z* restrains feasible differences in the mass defect of adjacent series peaks, denoted Δ*m*. The mass defect here refers to the deviation between an ion`s exact *m/z* value and its nearest integer [37]. For any monoisotopic chemical unit that could constitute a mass difference Δ*m/z*, bounds *γ*_*min*_ and *γ*_*max*_ for minimum and maximum differences in Δ*m* between a series peak *p*_*j,k*_ and another peak *p*_*j*+1*,k*_ can be determined by the mass defects of the isotopes of lowest mass for each of the elements contained in a unit. For example, and albeit lacking knowledge of the exact composition of a chemical unit but assuming only C, H, N, O, S, Cl and Br to be present, we can expect the value of Δ*m* between any two series peaks differing by Δ*m/z* to lie within [−0.0010 Δ*m/z*; 0.0078 Δ*m/z*]. The first factor *γ*_*min*_ is determined by the ratio of mass defect to atomic mass of ^79^Br, the second factor *γ*_*max*_ by the ratio for ^1^H. Factors for all of the other elements range in between these bounds. *γ* must be calculated over all chemical elements if no assumptions on the involved elements can be made. A mathematical definition of *γ*_*min*_ and *γ*_*max*_ is given in Additional file 1. Furthermore, one must account for the rounding involved in the calculation of mass defects: any Δ*m* along a series leading to mass defect values above 0.5 consequently wrap them to *Δm* − 1, whereas values below −0.5 convert to Δ*m* + 1. Thus, differences by Δ*m* must be adapted accordingly.

Similar to the bounds for Δ*m/z* and Δ*m*, deviations in retention time *RT* between adjacent series peaks must also be restricted in order to reflect reasonable chromatographic characteristics caused by repeated introduction of chemical units [29, 30]. On the one hand, Δ*RT*_*min*_ and Δ*RT*_*max*_ hence define minimum and maximum bounds for differences in *RT* from one peak *p*_*j,k*_ to its following tuple peak *p*_*j*+1*,k*_, respectively. On the other hand, changes in Δ*RT* across a series can be expected to be systematic [29, 30]. First, such changes in Δ*RT* from one pair of adjacent peaks in a tuple (*p*_*j,k*_, *p*_*j*+1*,k*_) to the next (*p*_*j*+1*,k*_, *p*_*j*+2*,k*_) must be smaller than a predefined value, denoted as ΔΔ*RT*. Second, cubic smoothing splines are fitted to model *RT* as a function of *m/z* in each series tuple [38]. Briefly, the model fit of each series as determined by the coefficient of determination (*R* ^2^) has to be above a certain threshold, using a preset smoothing parameter *λ* ≥ 0.

### Triplet detection

Constrained by the above outlined bounds for Δ*m/z*, Δ*m* and *ΔRT*, a first series detection stage uses *k*-dimensional (*k*-d) trees [39] as a metric data structure to enable a computationally fast extraction of peak 3-tuples which might be feasible sub-tuples of larger series tuples. In a *k*-d tree, each signal peak *x* from a LC-HRMS data set is represented by a vector $a_{x} \in {\mathbb{R}}^{4}$

1 $$a_{x} = \left( {m/z_{x} , \, \Delta m_{x} - \, \gamma_{min} m/z_{x} , \, \Delta m_{x} - \, \gamma_{max} m/z_{x} , \, RT_{x} } \right)$$

**Fig. 1:**
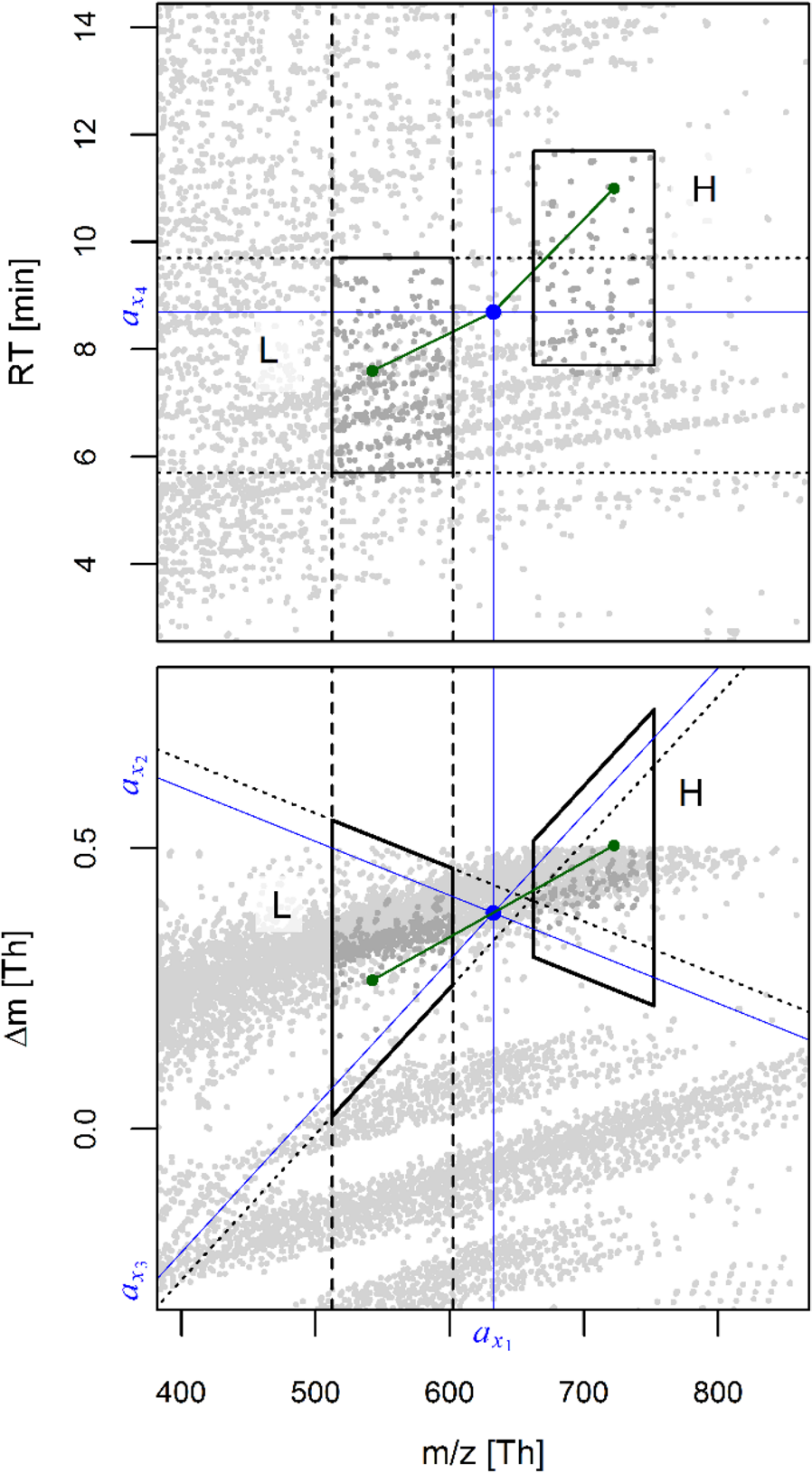
Exemplary subspace query (*black polygons*) for the detection of 3-tuples, centered at one peak (*blue point*) with one detected 3-tuple exemplified in *green*. Intersections of *blue lines* with axes indicate the values of the four elements of *a*_*x*_ for this peak. Moreover, *dashed lines* mark the *m/z* interval defined in Eq. (6). In turn, *dotted lines* in the top panel mark the *RT* interval of Eq. (9), whereas *dotted lines* in the *bottom panel* indicate the *upper* and *lower bound* of intervals related to mass defect differences from Eqs. (7) and (8), respectively

A geometrical depiction of the elements in *a*_*x*_ is given by the blue lines in Fig. 1. The second and third elements $a_{{x_{2} }}$ and $a_{{x_{3} }}$ transform the minimum and maximum change in mass defect with changing peak mass to a metric scale that can be represented in a *k*-d tree. In the latter, each tree node (alias peak) splits the space and the therein contained peaks into two partitions, using the peak with the median value for one of the elements in *a*_*x*_. The resulting two partitions are in turn split using the next element in *a*_*x*_, each by another median peak contained in those partitions (cp. numbered splitting planes in the TOC for an arbitrary example in ${\mathbb{R}}^{3}$). Starting with the first entry of *a*_*x*_ for the root node and recursively cycling over entries of *a*_*x*_ until partitions with a single peak (i.e., terminal nodes) are reached, a *k*-d tree supports fast range queries. These queries are conducted to extract all peaks from two subspaces of types *L* and *H* with lower and higher *m/z* repeatedly centered at each LC-HRMS peak. The extracted peaks are then successively recombined to screen for all unique peak combinations that can form 3-tuples in accordance with the above series definition and that include the current center peak as the second element of a triplet (cp. green and blue dots in Fig. 1). The peaks queried with these subspaces usually represent a minor fraction of all measured LC-HRMS peaks and the recombination to feasible triplets thus greatly improves over a check using all peaks. The mentioned subspace types L and H result from combining the intervals

2 $$I_{1} = [a_{{x_{1} }} + \Delta m/z_{min} ; a_{{x_{1} }} + \Delta m/z_{max} ]$$

3 $$I_{2} = [a_{{x_{2} }} - 2\varepsilon ;\infty ]$$

4 $$I_{3} = [ - \infty ;a_{{x_{3} }} + 2\varepsilon ]$$

5 $$I_{4} = \left[ {a_{{x_{4} }} + RT_{min} ;a_{{x_{4} }} + RT_{max} } \right]$$

6 $$I_{5} = [a_{{x_{1} }} - \Delta m/z_{max} ;a_{{x_{1} }} - \Delta m/z_{min} ]$$

7 $$I_{6} = [ - \infty ;a_{{x_{2} }} + 2\varepsilon ]$$

8 $$I_{7} = \left[ {a_{{x_{3} }} - 2\varepsilon ;\infty } \right]$$

9 $$I_{8} = [a_{{x_{4} }} - RT_{max} ;a_{{x_{4} }} - RT_{min} ]$$

via their Cartesian products to the queried subspaces

10 $$H = I_{1} \times I_{2} \times I_{3} \times I_{4}$$

11 $$L = I_{5} \times I_{6} \times I_{7} \times I_{8}$$

Intervals *I*_1_–*I*_4_ define bounds in each of the four dimensions of *a*_*x*_ for a subspace *H* succeeding the queried center peak. In contrast, intervals *I*_5_–*I*_8_ define a subspace *L* preceding the queried peak, as indicated by black lines and polygons in Fig. 1. Further details on how to account for the mentioned rounding issue of Δ*m* and to accelerate the computational retrieval of subspaces are provided in Additional file 2.

### Tuple recombination

The second stage successively combines the extracted 3-tuples to larger tuples. To this end, all pairwise combinations of tuples *x* and *y* from a set *S*_*n*_ which only differ in their first and last peak members, i.e.,

12 $$\left( {p_{1,x} , \ldots , \, p_{n - 1,x} } \right) = \left( {p_{2,y} , \ldots , \, p_{n,y} } \right)$$

or

13 $$\left( {p_{2,x} , \ldots , \, p_{n,x} } \right) = \left( {p_{1,y} , \ldots , \, p_{n - 1,y} } \right)$$

and conform to the above series definitions concerning *ε*, changes in *ΔRT* and *λ* are combined to a new *(n* + 1*)*-tuple in *S*_*n*+1_. After having formed all combinations from *S*_*n*_, the resulting tuples in *S*_*n*+1_ are in turn recombined to larger tuples in the next set *S*_*n*+2_. This is repeated until an empty set is reached, wherein each *n*-tuple is free to combine to several different (*n* + 1)-tuples. Therefore, a peak cannot be included more than once in one tuple, but several times in several different tuples. In every recursion, *n*-tuples which can be combined to at least one new (*n* + 1)-tuple in *S*_*n*+1_ are removed from *S*_*n*_; they otherwise remain in *S*_*n*_ or are discarded when ranging below a minimum user-defined length *n*_*min*_. Moreover, redundant sub-tuples which form by a regular omission of peaks in larger *n*-tuples of sets *S*_*n*≥5_ need to be filtered at each recursion.

### Series pairing

As mentioned, a peak may be a member of more than one series, as its containing 3-tuples might have been incorporated into several different larger tuples instead of a single one. To elucidate the underlying reasons for such ambiguities, all unique series pairs that intersect in at least one peak of a LC-HRMS sample were extracted and their properties characterized twofold.

On the one hand, the intersection angle *θ* was used to approximate in how far two series *x* and *y* of such a pair were superjacent in the plane of *RT* and *m/z*. *θ* is defined as

14 $$\cos \theta = \frac{{u_{x} \cdot u_{y} }}{{\parallel u_{x}\parallel \parallel u_{y}\parallel }}$$

In this equation, numerator and denominator state the dot product and the product of the Euclidean norm of vectors with scaled mean values

15 $$u_{x} = \left( {\frac{{\overline{{\Delta RT_{x} }} }}{{c_{\Delta RT} }},\frac{{\overline{{\Delta m/z_{x} }} }}{{c_{\Delta m/z} }}} \right)$$

of each series, respectively. Here, $c_{\Delta RT}$ and $c_{\Delta m/z}$ are the range of $\overline{\Delta RT}$ and $\overline{\Delta m/z}$ over all series. The smaller the value of *θ*, the more do two paired series overlie with each other in the *RT* vs. *m/z* plane. At *θ* = 0*π*, they are fully superjacent.

On the other hand, a self-organizing map (SOM) was used to visualize and cluster common properties among the paired series to explain differences in *θ* [40, 41]. Being an unsupervised learning strategy, SOMs allow the mapping of a large set of *m* multidimensional input vectors *v* = (*v*_1_ *,…,v*_*j*_ *,…,v*_*m*_) of series pair properties onto a smaller two-dimensional grid of SOM nodes. The SOM can then be selectively displayed for the mapped properties; similar properties are herein mapped to close regions in the SOM while different ones are rather separated. In the given case, each input vector of series pair properties

16 $$v_{j} = \left( {\frac{{\overline{{\Delta RT_{x} }} }}{{\hat{c}_{\Delta RT} }},\frac{{\overline{{\Delta m/z_{x} }} }}{{\hat{c}_{\Delta m/z} }},\frac{{\overline{{\Delta RT_{y} }} }}{{\hat{c}_{\Delta RT} }},\frac{{\overline{{\Delta m/z_{y} }} }}{{\hat{c}_{\Delta m/z} }}} \right)$$

contains the mean values of *m/z* and *RT* differences present in paired series *x* and *y*, arranged by $\overline{{\Delta m/z_{x} }} \ge \overline{{\Delta m/z_{y} }}$ and $\hat{c}_{\Delta RT}$ and $\hat{c}_{\Delta m/z}$ representing the mean expected measurement uncertainties of Δ*RT* and Δ*m/z*, respectively. Based on these properties, an intersection angle *θ* can be calculated for each SOM node via Eq. (14) to estimate the superjacency of series mapped onto it. Further information on the training and quality of the SOM is provided in the Additional file 3. The SOM calculations were conducted with the R *kohonen* package, parameterized as listed in the Additional file 4: Table S1 [42].

### Sampling and analysis

Evaluation was carried out on 24 h flow-proportional samples taken from the effluent of ten Swiss sewage treatment plants in February 2010, as used and detailed in Schymanski et al. [16]. In short, a sample volume of 0.25 L was each pH-adjusted, filtered, spiked with 103 isotope-labeled standards and enriched via a mixed-bed solid-phase extraction. After basic/acidic extraction, further enrichment under a nitrogen gas stream, reconstitution with HPLC water to 1 mL and a second filtering step, a final aliquot of 20 *μ*L was analyzed with HPLC-ESI-HRMS. The chromatographic step comprised Waters XBridge C18 columns (Milford, USA) and a water/methanol gradient at a flow rate of 200 *μ*L/min generated by a Rheos 2200 low pressure mixing pump (Flux instruments, Basel, Switzerland). A Q-Exactive (Thermo Fisher Scientific, San Jose, USA) was used for full-scan mass spectrometric analysis at a resolution of 140,000 at *m/z* = 200, following electrospray ionization in each positive and negative modes (spray voltage +4 and −3 kV, respectively; 350 °C capillary temperature). A blank measurement was run prior to each block of positive and negative sample aliquots, respectively. The data files are openly accessible via the *MassIVE* repository [43].

### Data processing

LC-HRMS full-scan data were centroided and converted to open mzXML format files with ProteoWizard (version 3.0.7162) [44, 45]. All downstream analysis was then run in the R statistical environment [46]. Utilizing the R package *enviPick* (version 1.2) [47], ion chromatograms were extracted in each file and each extracted chromatogram screened for signal peaks, with parameters listed in the Additional file 5: Table S2. Upon peak-picking, series were detected with the above outlined algorithm, as parameterized in Additional file 6: Table S3. For each peak being part of a series, both a blank subtraction and a deisotoping was run with the *enviMass* v3.1 [48] and the *nontarget* v1.9 [49] packages, respectively (see Tables S4 and S5 for parameters in Additional files 7 and 8). In the first case, a peak-centered *RT* and *m/z* window was checked for each sample peak to not contain raw blank data points higher than 0.1 times the maximum sample peak intensity to certify its presence in the effluent. A majority rule, i.e., a fraction of ≥0.5 peaks per series, was used for a final assignment of a series to be of blank origin. For deisotoping, a comparison with quantized simulation data enabled a grouping of the isotopologue peaks of an unknown compound, within given measurement uncertainties. The peaks in the individual isotopologue groups of each series peak were then ranked by increasing *m/z*. A series was assumed to be monoisotopic if the most frequent rank over all peaks in a series equaled 1.

## Results and discussion

### Series inventory and recovery

On average (±standard deviation, SD), 21,153 ± 3052 and 10,418 ± 831 peaks were picked from the LC-HRMS measurements of the 10 STP samples in positive and negative ionization modes, respectively (Table S6, Additional file 9). A substantial mean fraction of 0.37 ± 0.09 of these peaks could be assorted into series for the positive mode, whereas a smaller and less variant fraction of 0.13 ± 0.03 was assorted in the negative mode. Only few of these detected series are likely caused by chance alone, as in fully unrelated sets of peaks. As estimated by additional randomization experiments in Table S7 of Additional file 10, false discovery rates amounted to much smaller mean fractions of 0.02 ± 0.01 and below for the positive and negative ionization modes, respectively. Furthermore, overall numbers of peaks assigned to series were strongly correlated with the total number of picked peaks in a STP sample, although series peaks dominated the measured set of picked peaks at only one location (STP ID 8, positive mode). Series counts were in turn correlated with the fraction of series peaks for both ionizations although the length of individual series varied greatly, from five and up to 30 peaks. Notably, series counts were often on the same order as the peak counts of which they were comprised, for reasons discussed in the next section. Overall, 7576 ± 4222 and 1018 ± 494 series were detected in positive and negative modes, respectively. The large SD was mainly driven by one STP (ID 8, Table S6).

To test the presented algorithm, a ground truth set of eight known HS compounds was utilized. These compounds had each at least five of their homologues tentatively identified in a majority of the discussed STP samples in a suspect screening campaign conducted by Schymanski et al. [16]; they consisted of the surfactants LAS, SPAC, DAT, STAC, C_12_-AES, C_13_-AES, SAS and PEG as listed in Table S6 of the named study. In line with this previous study, the full peak series of the four surfactants SPAC, STAC, C_12_-AES and PEG were consistently recovered in all ten STP samples by our algorithm. The peak series of the remaining four HS compounds were recovered in nine (DAT), four (SAS) and three (LAS, C_13_-AES) samples. In all other cases, series could not be recovered because either not all series peaks were consistently picked at lower intensities (40% of cases) or had partly erratic *RT* behavior (60% of cases). The algorithm thus successfully retrieved all continuous HS peaks with systematic *RT* differences among the individual homologues. Furthermore, homologue peaks in addition to those individually screened in the named study were detected in at least six cases. In another six cases, some of the HS peaks were also integrated into series other than those covered in the named study, hence complementing the previous suspect screening approach (cp. Figures S2 and S3 in Additional files 11 and 12).

Moreover, much lower series counts were observed in the two blank measurements. Only few of the STP sample series were conversely removed via majority voting during the blank subtraction step, i.e., series fractions of 0.10 ± 0.06 (positive ionization mode) and 0.07 ± 0.03 (negative ionization mode). Their absolute numbers correlated negatively with the total number of picked peaks in a sample, which may be explained by varying degrees of matrix suppression of blank signals in more complex samples. Of the remaining non-blank series, fractions of 0.46 ± 0.13 (positive) and 0.27 ± 0.08 (negative) series contained sporadic peaks which did not pass the blank subtraction individually. This may be attributed either to false detection of series comprising sporadic peaks also present in the blank or to uncertainties in the blank subtraction for an existing series. Deducing from the above mentioned randomization experiment (Table S7 of Additional file 10), we expect the first case to be less frequent than the second. For the latter, running the blank subtraction after peaks were assorted into series instead of before can help avoid sporadic series gaps which impede series detection. On the other hand, removing all sample series with sporadic blank peak assignments would overestimate counts of such sample blank series by an order of magnitude as compared to series counts found in the blank measurements. Similar uncertainties existed for the filtering of monoisotopic series, with their counts listed in column 8 of Table S6 in Additional file 9. Fractions of 0.72 (positive) and 0.46 (negative) of monoisotopic series contained infrequent peaks with masses suggesting a non-monoisotopic composition (i.e., with *m/z* rank > 1), which is in line with the false positive rates of isotopologue grouping. Using ensembles of peaks in each series after the series detection step instead of an earlier deisotoping based on singular peaks might thus improve deisotoping.

### Series computation

The restrictions for Δ*RT*, Δ*m/z* and Δ*m* localized at each center peak decrease the computational burden of detecting meaningful 3-tuples. The total number of all possible 3-tuple peak combinations in samples thereby reduced by around seven orders of magnitude to averages of 4.3 × 10^5^ and 1.0 × 10^5^ 3-tuples for the positive and negative ionization mode, respectively. Of these triplets, fractions of only 0.13 (positive) and 0.08 (negative) passed into 4-tuples through pairwise combinations; passed fractions then strongly increased towards higher *n*-tuple combinations. At this stage, fractions of up to 0.14 4-tuple combinations could be excluded for having erratic changes in Δ*RT*, which then dropped mostly to zero for series with length *n* ≥ 5. Additional exclusion criteria such as the similarity of chromatographic peak shapes or the distribution of Δ*m/z* and intensity in a series may be approached in future versions. Overall, the computation time for series detection never exceeded 4.1 min per sample on a standard computer, including parsing of results, and decreased rapidly with the number of detected triplets. For negative mode samples, computation time was hence below 0.5 min (Windows 7, R version 3.1.3, 2.2 GHz Intel core i7-4702 MQ processor, single-core usage, 32 GB RAM, 64 bit).

### Superjacent series

**Fig. 2:**
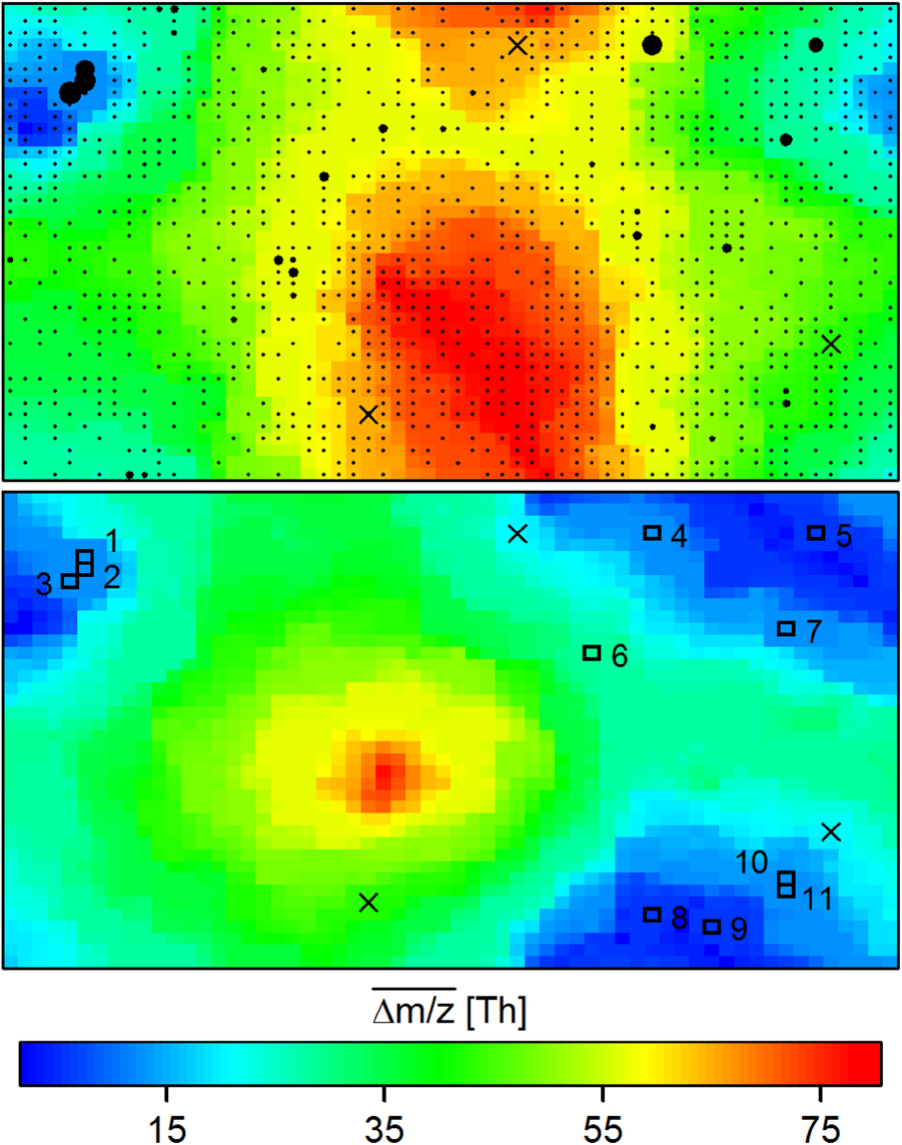
SOM for series pairs from STP sample with ID = 1, positive ionization mode. *Coloring* of the *top* and *bottom panels* show $\overline{\Delta m/z}_{x}$ and $\overline{\Delta m/z}_{y}$ values at the SOM nodes, respectively. *Sizes* of the *black dots* in the *top panel* indicate frequencies of monoisotopic series pairs mapped onto the nodes. In turn, *black squares* in the *bottom panel* either indicate the three nodes with highest frequencies at low intersection angles (*θ* < 0.08*π, nodes 1–3*) or nodes with highest mapping frequencies containing 50% of all monoisotopic series pairs at larger intersection angles (*θ* ≥ 0.08*π*, *nodes 4–11*). The series mapped onto the latter nodes 4–11 are shown in Fig. 3. Moreover, crosses highlight the mapping nodes of the unknown superjacent series shown in Figure S6 (Additional file 15)

The incorporation of a single peak into different series was common to all samples and ionization modes. Dominant mean fractions of 0.99 ± 0.01 (positive) and 0.96 ± 0.02 (negative) series thus shared peaks with other series, for reasons elucidated further below. Often, much more than one peak sharing existed per series, leading to a multitude of series pairs with at least one peak in common (last two columns of Table S6 in Additional file 9). A SOM was hence trained for one STP sample (positive ionization, ID = 1 in Table S6) to map and cluster properties of series pairs that can explain such peak sharing for different intersection angles between the paired series. The resulting SOM with node values for $\overline{{\Delta m/z_{x} }}$ and $\overline{{\Delta m/z_{y} }}$ in the top and bottom panels is shown in Fig. 2. To recall, $\overline{\Delta m/z}$ is the mean Δ*m/z* in a series; concomitant distributions of $\overline{\Delta RT}_{x}$ and $\overline{\Delta RT}_{y}$ across SOM nodes can be found in the Additional file 13: Figure S4.

Based on the SOM, several observations can be made. First, although series pairs with a wide array of different $\overline{\Delta m/z}$ and $\overline{\Delta RT}$ values exist, many pairs nevertheless cluster at certain nodes (black dots in Fig. 2 and Figure S4 of Additional file 13). In fact, just 13% of the nodes are able to summarize 90% of the pairs. This indicates that dominant patterns in series properties can account for significant proportions of series being paired with other series. Second, the contribution of non-monoisotopic series herein is noteworthy, affecting as much as 42% of the pairings. Third, a majority of series pairs intersect at low angles *θ* and are therefore largely superjacent, i.e., they are similarly positioned in the *RT* and *m/z* plane. Using a histogram-derived threshold of *θ* < 0.08*π*, this affects a predominant fraction of 0.81 series pairs in the considered STP sample (Additional file 14: Figure S5; cp. last column of Table S6 in Additional file 9 for fractions in other STPs). The concomitant SOM nodes onto which such superjacent pairs map are shown in white in Additional file 16: Figure S7. In these SOM regions, nodes with both series in a pair having $\overline{\Delta m/z}$ ≈ 14.016 are most frequently used for mapping (nodes 1–3 in Fig. 2 and S4 of Additional file 13). Based on an inspection of the LC-HRMS data, it can be concluded that these superjacent series frequently result from close-eluting isobaric peaks. If overlapping in the Δ*RT* window of different tuples, isobaric peaks can cause an exponential increase in the number of possible combinations for forming series from these tuples. For example, 2^*n*^ series combinations of comparable $\overline{\Delta m/z}$ arise for *n* pairs of isobaric peaks each located at different *m/z* values. Isobaric peaks from homologue isomers are indeed common and may require additional analytical separation to be extractable as fully non-superjacent series [15, 50]. One confirming example known to have isobaric peaks from different isomers of homologues differing by CH_2_ at $\overline{\Delta m/z}$ ≈ 14.016 is provided for the identified SPAC surfactant in Figure S2 of Additional file 11, albeit for the negative ionization mode.

Another less frequent reason for superjacent series was the sporadic occurrence of missing peaks in otherwise continuous series, e.g., at series ends with diminishing measurement intensities. As a result, closely superjacent series with $\overline{\Delta m/z}$ being multiples of each other are detected. Because the affected series are no strict subsets of each other, they cannot be eliminated during the removal of sub-tuples at the end of the second stage of the algorithm. An aggravated example for illustrating superjacency caused by such series gaps is provided in Figure S6 of the Additional file 15. To clarify, $\overline{\Delta m/z}$ values being multiples of each other can also arise for differently charged adducts of the same homologue series; these multiples are however not superjacent and can thus be distinguished. Similarly, the different series of the different isotopologues of a homologue compound are unlikely superjacent but rather parallel in orientation in the *m/z* vs. *RT* plane.

### Meshed series

**Fig. 3:**
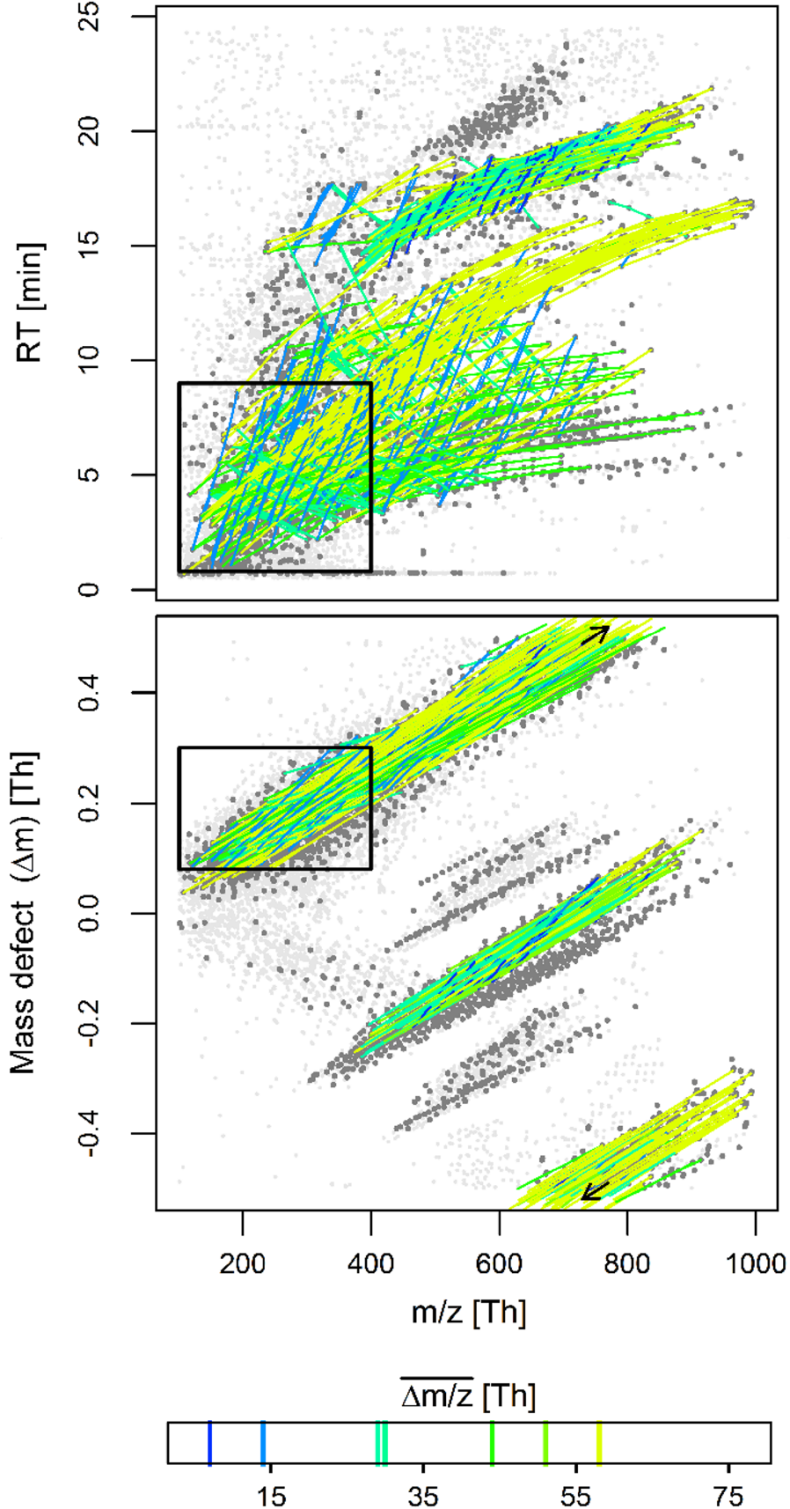
Meshed series pairs from nodes 4–11 of Fig. 2, *colored* by their series mass differences $\overline{\Delta m/z}$. *Light gray* points show all picked peaks whereas only those depicted in *dark gray* are part of any series. *Bold rectangles* indicate the zoom area for Figure S3 (Additional file 12) to depict the PEG-related series

A notable 19% of series pairs were not superjacent (highlighted by the heat colors in Figure S7 of Additional file 16), but instructively arranged. For closer inspection, the set of most strongly clustered monoisotopic series pairings at intersection angles *θ* ≥ 0.08*π* was selected from the SOM (black squares 4–11 in Fig. 2 and S4 of Additional file 13). The chosen series are in turn plotted in Fig. 3 and comprise seven distinct values in $\overline{\Delta m/z}$.

One first group of interrelated series embraces $\overline{\Delta m/z}$ values of 14.016, 44.026, 30.011 and 58.042 *Th*, with multiple pairings between these values. The co-occurrence of these values can be illustrated by using a subset of series related to the known PEG surfactant, shown in Figure S3 of Additional file 12. Therein, the first two values stem from Ethoxylate (C_2_H_4_O_1_) and possibly Alkyl (CH_2_) homologue units of variable length, with the first identified as part of the known PEG series (black triangles in Figure S3). Confirmingly, co-occurrence of both units has also been reported for homologues found elsewhere in STP effluents [12, 15, 51]. With (a) both units coexisting at all their differing lengths and (b) varying *RT* increases for both the resulting chains, a mesh-like orientation of these series in the *m/z* vs. *RT* plane arises. In addition, the mutual orientation of both series types allows for further cross-meshing, formed by a subtraction (C_1_H_2_O_1_) and a sum (C_3_H_6_O_1_) of the former two homologue units. This overall hypothesis is also in agreement with observed mass defect differences Δ*m*, which are smaller for higher O/C ratios in these four series types (lower panel of Figure S3).

A second major group of interrelated series pairs extracted from the concerned SOM nodes comprises $\overline{\Delta m/z}$ values of 7.008, 29.021, 51.034 and 58.042 *Th*. This second group is likely a result of adduct formation at *z* = 2, considering (a) concomitant mass defect differences (cp. lower panel of Fig. 3), (b) the first two values being halves of the above discussed $\overline{\Delta m/z}$ values of 14.016 and 58.042 *Th* and (c) the latter two values formable by multiples and subtractions among the former two.

Several implications related to the outlined meshing must further be stressed. First, series meshing does not only provide complementary information, but can also prevent false conclusions. That is, a $\overline{\Delta m/z}$ value of 58.042 *Th* may as well suggest the occurrence of a propylene oxide unit instead of a sum of two different units—and propylene units are known to exist for homologue series [52]. As a matter of fact, other series with $\overline{\Delta m/z}$ = 58.042 *Th* not participating in any meshing occur in the very same STP sample, but have yet to be chemically identified. Second, negative *RT* differences (Δ*RT*_*min*_ < 0) can arise for peak series formed by subtractions in cross-meshing (such as the one with $\overline{\Delta m/z}$ = 30.011 *Th* in Figure S3 of Additional file 12), even when *RT* is expected to increase with the length of the underlying chemical homologue chains (as exemplified for another unknown series with the same mass difference in Figure S2 of Additional file 11). Third, cross-meshed series with $\overline{\Delta m/z}$ values not matching any molecular formula can arise if the atoms of the homologue units do not form subsets. In the above first example group, C_2_H_4_O_1_ minus CH_2_ equals C_1_H_2_O_1_; however, a hypothetical C_2_H_4_O_1_ minus CF_2_ would in contrast not suggest a valid molecular formula. Fourth, meshed series may have fixed sets of $\overline{\Delta m/z}$ values but likely a more variable set of $\overline{\Delta RT}$ values. In the SOM, this latter variation is covered by several mapping nodes, which should nonetheless be close to each other in the SOM if the topological continuity holds (cp. black squares 10 and 11 in the bottom panel of Fig. 2 for two such adjacent nodes). Finally, the complexity of series meshing will rise with the number of homologous chains per compound. Even for the discussed example, further additions and subtractions from cross-meshing of (CH_2_)_2_ and (C_2_H_4_O_1_)_2_ units exist, but these were less frequent and hence not selected from the SOM here.

### STP comparison

**Fig. 4:**
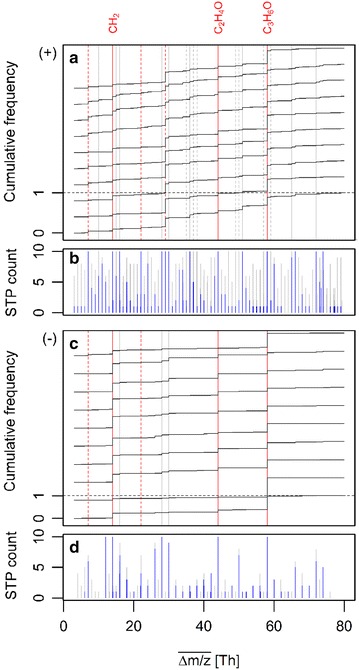
Relative cumulative frequency of $\overline{\Delta m/z}$ values for all blank-subtracted series detected in positive (**a**) and negative (**c**) ionization modes, stacked top-down for individual STP samples in order of their IDs 1–10 (cp. Table S6 in Additional file 9). *Solid red lines* indicate masses of three common homologue units at *z* = 1, *red dashed ones* at *z* = 2. *Solid gray lines denote* $\overline{\Delta m/z}$ values of possible multiples, additions or subtractions thereof. *Gray dashed lines* indicate isotopologue shifts of some of these masses, equal to ^12^C vs. ^13^C mass transitions. Moreover, *gray bars* in **b** and **d** show STP counts from a moving $\overline{\Delta m/z}$ window (±5 μ) over all stacked distributions for the positive and negative mode, respectively. In contrast, *blue bars* were derived after omission of non-monoisotopic series

To complement the above exemplification of series patterns based on only a single STP sample, Fig. 4 ultimately stacks the $\overline{\Delta m/z}$ distributions of all blank-corrected series for every STP at both ionization modes (panels A and C, black lines) and filters for $\overline{\Delta m/z}$ values prevalent across STPs (panels B and D, gray bars). Noteworthy, a multiplicity of $\overline{\Delta m/z}$ values exist, many of which are highly conserved across the different STPs, although at different frequencies and with less diversity in the negative than in the positive ionization mode. Among the most frequent, especially in the negative mode, are the three discussed values of $\overline{\Delta m/z}$ = 14.016, 44.026 and 58.042 *Th*, partly corresponding to alkyl, ethoxylate and possibly propylene oxide units (red solid lines) [16]. The larger frequency of the latter again suggests another origin than the mere addition of the former two units as presented above, both at charges *z* = 1 and *z* = 2. Other than that, a large but still incomprehensive fraction of the remaining $\overline{\Delta m/z}$ values might be annotated via either charge- or gap-related multiples or additions/subtractions of these three units, albeit tentatively until identified as such (red dashed and gray bars). Moreover, seven of the most ubiquitous yet low-frequent $\overline{\Delta m/z}$ values among STPs in positive mode almost disappear when non-monoisotopic series are excluded from the cumulative frequency analysis (gray dashed lines, blue bars). Their values occur around major non-affected ones at mass differences equal to those between ^12^C and ^13^C and may involve series of different isotopologues of different carbon-rich members of homologue series. However, without further identification attempts—which can now gain from additional information on series meshing and $\overline{\Delta m/z}$ co-occurrence across STPs—such annotations remain largely speculative. Given the prevalence of some $\overline{\Delta m/z}$ values, detected series may nonetheless be engaged to cluster different STPs, to quantify the ubiquity of series across STPs or to find similarities of unpaired series arising from, e.g., transformations by a second SOM training.

## Implementation

The outlined algorithm is freely available as function *homol.search()* in the R package *nontarget* [49] and accessible through a web-interface at www.envihomolog.eawag.ch [53]. With the package, parameters Δ*m/z*_*min*_, Δ*m/z*_*max*_, Δ*RT*_*min*_, Δ*RT*_*max*_, ΔΔ*RT, n*_*min*_ *, ε, λ, R* ^2^ and the involved chemical elements can all be user-defined (cp. Table S3 in Additional file 3). Optionally, restrictions for Δ*m/z* can be included for a more targeted series detection or to confine the numbers of computed series in samples with even higher HS contents, e.g., oil extracts. Spline smoothing can be disabled and changes in Δ*RT* increased to comprise series with erratic *RT* behavior, although this will almost certainly trigger more false positive series as a trade-off. Series results can finally be tagged to adduct and isotopologue groups with the package to derive component peak sets; the package documentation contains instructive script examples for executing all functions with an exemplary list of peaks. As complement, the web-interface facilitates series detection and data handling for non-programmers and allows a fully interactive visualization, filtering and export of results. Extensive clustering of series pairs such as the proposed SOM is not scope of the package or web-interface.

## Conclusion

Given the large throughput in LC-HRMS experiments, a visual detection of systematic signal patterns to pinpoint the presence of unknown homologous compounds from the accumulated data is futile. Hence, an untargeted yet efficient bottom-up computation of picked peak series with systematic differences in mass and retention time is presented and evaluated. With just a minimum of prior information on expected homologous compounds to confine this detection, the presented algorithm will reveal series regardless of their specific ionized species, certain modifications during ionization or nonlinear *RT* properties. While coping with variable measurement uncertainties, the algorithm enables the detection of low-frequent and low-intense series even in complex matrices if series peaks are properly picked and reach a minimum but adjustable series length. Furthermore, non-random inclusion of peaks into different series proved useful to discern possible ambiguities in assigning peaks to series and to identify series meshing caused by homologues with more than a single variable chemical unit. The detected series are highly beneficial as they facilitate subsequent identification efforts, can lead to a substantial data reduction and provide additional nontargeted statistics to compare different samples, amongst others. Future research might implement gap-tolerant versions of the proposed algorithm and further data mining to automatize the digestion of the wealth of complex series interrelations.

## Additional files

**Additional file 1.** Definition of bounds for mass defect differences.

**Additional file 2.** Mass defect rounding issue and computational acceleration.

**Additional file 3.** SOM training details.

**Additional file 4.** SOM training parameters.

**Additional file 5.** Parameters for chromatogram extraction and peak picking.

**Additional file 6.** Parameters for series detection.

**Additional file 7.** Blank subtraction parameters.

**Additional file 8.** Isotopologue grouping parameters.

**Additional file 9.** STP peak and series detection characteristics.

**Additional file 10.** STP series detection characteristics following randomization.

**Additional file 11.** LC-HRMS peaks and series of the SPAC surfactant.

**Additional file 12.** Meshed structure of PEG and other yet unidentified LC-HRMS peak series.

**Additional file 13.** SOM results on *ΔRT* for paired series detected in the Affoltern STP effluent, Switzerland.

**Additional file 14.** Histogram of intersection angles between paired SOM series.

**Additional file 15.** Superjacent series exemplification.

**Additional file 16.** Intersection angle θ of SOM nodes.

## Abbreviations

HPVC: high production volume chemicals
HRMS: high-resolution mass spectrometry
HS: homologue series
LC: liquid chromatography
NN: nearest neighbor
RT: retention time
SOM: self-organized map
STP: sewage treatment plant
