# Supplementary material for: Nontargeted homologue series extraction from hyphenated high resolution mass spectrometry data
Source: J Cheminform. 2017 Feb 23;9:12. doi: 10.1186/s13321-017-0197-z (PMC5323340; doi:10.1186/s13321-017-0197-z)
Supplement: Supplementary file 1 — Additional file 1. Definition of bounds for mass defect differences. [file 13321_2017_197_MOESM1_ESM.docx]

The minimum and maximum allowable bounds *γ_min_* and *γ_max_* in mass defect differences Δ*m* between series peaks can be determined by the mass defects of isotopes of the elements contained in the underlying chemical unit of monoisotopic composition. More precisely, let *M_i_^*^* and *m_i_^*^* denote the mass defect and atomic mass of the isotopes of lowest mass for each element *i* of a total of *n* elements contained in a chemical unit, respectively. Then the minimum and maximum bounds can be defined as

$\gamma_{min}=\min_{i\in\{1,\ldots,n\}} \left( {M_{i}^{*}}/{m_{i}^{*}} \right)$ (1)

and

$\gamma_{max}=\max_{i\in\{1,\ldots,n\}} \left( {M_{i}^{*}}/{m_{i}^{*}} \right)$ (2)

Consequently, the resulting range of permissible changes in Δ*m* for a change in Δ*m/z* is restricted by

$\gamma_{min}\leq\frac{d\Delta m}{d\Delta m/z}\leq\gamma_{max}$ (3)

For instance, the difference Δ*m* in a series is bound by [-0.0010Δ*m/z*; 0.0078Δ*m/z*] for any unknown chemical unit assumed to only contain the elements C, H, N, O, S, Cl and Br. The first factor *γ_min_* = -0.0010 is the *M^*^* to *m^*^* ratio of ^79^Br, the second factor *γ_max_* = 0.0078 the ratio for ^1^H. Factors for all the other mentioned elements range in between these bounds. *γ* can be calculated over all chemical elements if no assumptions on the elements present in the chemical units can be made.
