# Supplementary material for: Nontargeted homologue series extraction from hyphenated high resolution mass spectrometry data
Source: J Cheminform. 2017 Feb 23;9:12. doi: 10.1186/s13321-017-0197-z (PMC5323340; doi:10.1186/s13321-017-0197-z)
Supplement: Supplementary file 2 — Additional file 2. Mass defect rounding issue and computational acceleration. [file 13321_2017_197_MOESM2_ESM.docx]

Because of the rounding involved in its definition, consecutive mass defect differences Δ*m* along a series cannot lead to mass defects above 0.5 but instead wrap to *Δm-1*, whereas values below -0.5 convert to Δ*m+1*. Thus, differences by Δ*m* must be adapted accordingly and require the definition of four additional subspaces to be queried for each peak. Two of these subspaces termed *L´* and *L´´* complement subspace *L* which precedes each queried center peak to find triplet peaks with lower *m/z* values. The other two complement subspace *H* which succeeds a center peak to find triplet peaks with higher *m/z* values; these are termed *H´* and *H´´*. Based on the intervals *I_1_* to *I_8_* given in the main manuscript, these additional subspaces are formally defined by the following Cartesian products:

$H^{'}=I_{1}\times{(I}_{2}+[1;0])\times{(I}_{3}+[0;1])\times I_{4}$ (1)

$H^{''}=I_{1}\times{(I}_{2}-[1;0])\times{(I}_{3}-[0;1])\times I_{4}$ (2)

$L'=I_{5}\times{(I}_{6}+[0;1])\times(I_{7}+[1;0])\times I_{8}$ (3)

$L''=I_{5}\times{(I}_{6}-[0;1])\times(I_{7}-[1;0])\times I_{8}$ (4)

Because of translations with [*1;0*] and [*0;1*] in the above equations, the primed subspaces *L´,* *L´´* and *H´,* *H´* have the same shape but a different position in the queried space. The primed and unprimed query subspaces are exemplified by black polygons in the below Figure S-1. In practice, translated subspaces need only be queried if they intersect with the feasible subspace of peak data within *-0.5 ≤ Δm ≤ 0.5*.

The successive recombination to feasible triplets can be built on a linear search over all peaks queried from subspace types *L* and *H*, ordered by their absolute Δ*m/z* relative to the center peak. This recombination can be computationally facilitated in two ways. First, the step size from one center peak to the next, i.e., the concomitant change of *a_x_*, can be kept small. The amount of peaks that then leave and enter the re-centered subspaces is kept small, too. As a consequence, most queried peaks are already partially ordered, accelerating their reordering in Δ*m/z* relative to the next center peak [1]. Second, branches in the *k-*d tree which were fully enclosed in the subspace of one center peak and which are fully enclosed for the next need not be fully traversed again for the query. Here, the nearest neighbor (NN) algorithm with an Euclidean distance was used to construct a path for re-centering, after normalization of each dimension by its range (below Figure S-1, red line) [2].


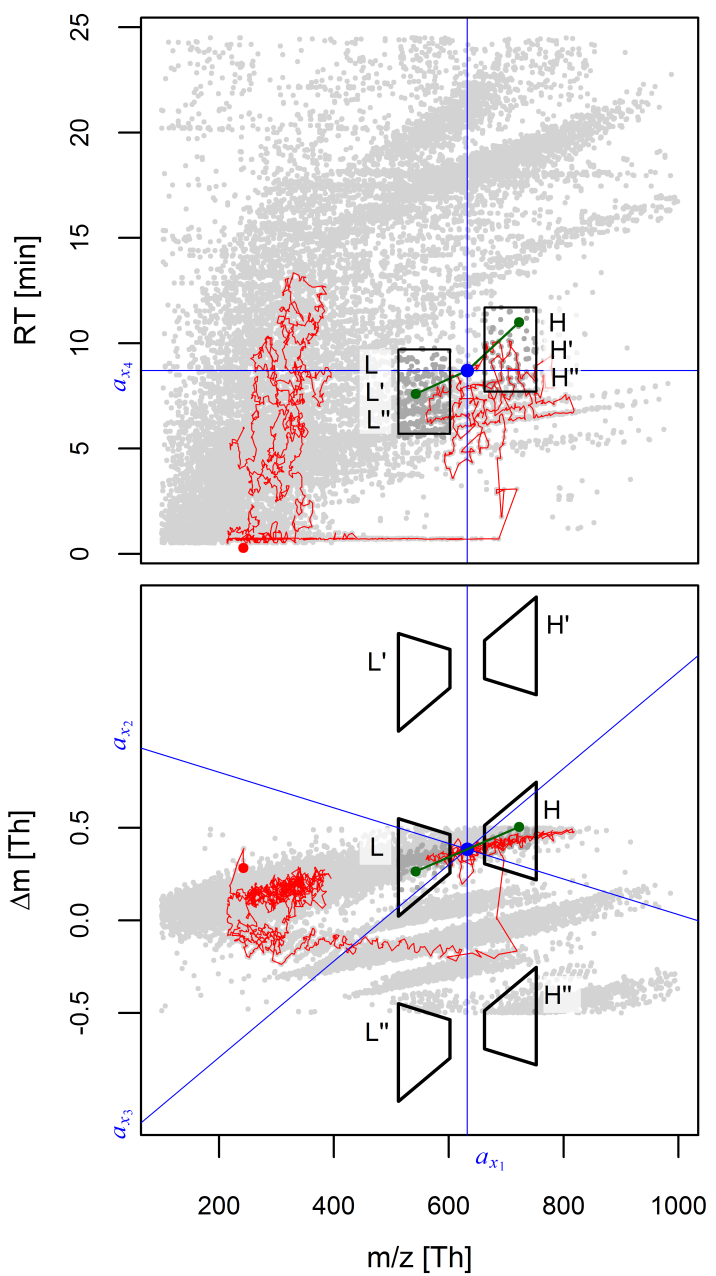


**Figure S-1.** Exemplary subspace query (black polygons) for the detection of 3-tuples, centered at one peak (blue point) after 2499 re-centering steps along the NN path (red line, random starting point shown as red dot) through the picked peaks (gray points). Intersections of blue lines with axes indicate the values of the four elements of *a_x_* for the centered peak. One detected *3-*tuple is exemplified in green, with the center peak as its second element. Note that all unprimed and primed lower (L) and higher (H) query subspaces are stacked in the top panel.

**References**

1. Singleton R. An efficient algorithm for sorting with minimal storage: Algorithm 347. Comm ACM. 1969;12(3):185-187.

2. Johnson DS, McGeoch LA. The traveling salesman problem: A case study in local optimization.

Local search in combinatorial optimization. Chichester, UK; 1997;1:215-310.
