# Supplementary material for: Nontargeted homologue series extraction from hyphenated high resolution mass spectrometry data
Source: J Cheminform. 2017 Feb 23;9:12. doi: 10.1186/s13321-017-0197-z (PMC5323340; doi:10.1186/s13321-017-0197-z)
Supplement: Supplementary file 3 — Additional file 3. SOM training details. [file 13321_2017_197_MOESM3_ESM.docx]

For training a SOM, vectors from *v* are sequentially used to update both their best-matching node *d* and other nodes in a shrinking neighborhood of *d*, with matching based on Euclidean distances. At each such sequential iteration *t* with a current *v_j_*, the update of each node vector *W_i_* is calculated via

$W_{i}\left( t+1 \right)=W_{i}(t)+\Phi(d,i,t)\alpha(t)[v_{j}-W_{i}\left( t \right)]$ (1)

where *α(t)* denotes a learning rate which here declines linearly over the iterations. Similarly, $\Phi(\ldots)$ defines a shrinking rectangular neighborhood around *W_d_* ; *W_i_* outside of this neighborhood remain unchanged. Overall, the full data set *v* was presented multiple times to the SOM for training, which was randomly initialized from *v* for *t=0* with a toroidal rectangular grid.

The quality of the trained SOM was assessed by the quantization and topological errors *E_q_* and *E_t_*, respectively


[1,2]. The first error is the mean distance of all vectors in *v* to their best matching node in the final map, expressed in terms of unscaled $\bar{\Delta RT}$ and $\bar{\Delta m/z}$ values. Thus, *E_q_* states the accuracy with which the input vectors are represented by the SOM. For the series pairs of the selected STP sample with ID=1, this first mapping error thus amounted to *E_q_(*$\bar{\Delta m/z}$*) = 0.02 Th* and *E_q_(*$\bar{\Delta RT}$*) = 0.12 min*. Additionally, the second metric *E_t_* measures the continuity of the mapping by the distance between the best and second best matching unit averaged over all vectors in *v* and expressed in terms of grid coordinates. This topological error amounted to *E_t_ = 1.6* for the discussed STP sample projection of series pairs.

*References*

1. Kiviluoto K. Topology preservation in self-organizing maps. Helsinki University of Technology; 1995.

2. Kohonen T, Schroeder M, Huang T, Maps S-O. Springer-Verlag New York. Inc, Secaucus, NJ. 2001;43.
