# Supplementary material for: Nontargeted homologue series extraction from hyphenated high resolution mass spectrometry data
Source: J Cheminform. 2017 Feb 23;9:12. doi: 10.1186/s13321-017-0197-z (PMC5323340; doi:10.1186/s13321-017-0197-z)
Supplement: Supplementary file 4 — Additional file 4. SOM training parameters. [file 13321_2017_197_MOESM4_ESM.docx]

Table S1. Parameters used for SOM training with the R *kohonen* package.

| **Parameter** | **Article symbol** | **Value** |
| --- | --- | --- |
| - | $\hat{c}_{\Delta RT}$ | *0.1* |
| - | $\hat{c}_{\Delta m/z}$ | *0.001* |
| xdim | *-* | *50* |
| ydim | *-* | *30* |
| rlen | *-* | *10.000* |
| alpha | *α* | *5x10^-3^ to 1x10^-5^* |
| radius | *-* | *45* |
| init | *-* | *- (default)* |
| toroidal | *-* | *TRUE* |
| n.hood | *-* | *square* |
