# Supplementary material for: Nontargeted homologue series extraction from hyphenated high resolution mass spectrometry data
Source: J Cheminform. 2017 Feb 23;9:12. doi: 10.1186/s13321-017-0197-z (PMC5323340; doi:10.1186/s13321-017-0197-z)
Supplement: Supplementary file 5 — Additional file 5. Parameters for chromatogram extraction and peak picking. [file 13321_2017_197_MOESM5_ESM.docx]

Table S2. Parameters used for peak picking with the R *enviPick* package, functions *mzagglom()*, *mzclust()* and *mzpick(),* respectively. See package manual for detailed parameter descriptions.

| **Parameter** | **Value** |
| --- | --- |
| dmzgap | *>3.5* |
| ppm | *TRUE* |
| drtgap | *300 [seconds]* |
| minpeak | *4* |
| maxint | *1x10^7^* |

| **Parameter** | **Value** |
| --- | --- |
| dmzdens | *3.5* |
| ppm | *TRUE* |
| drtdens | *60 [seconds]* |
| minpeak | *4* |
| maxint | *1x10^7^* |

| **Parameter** | **Value** |
| --- | --- |
| minpeak | *4* |
| drtsmall | *20 [seconds]* |
| drtfill | *10 [seconds]* |
| drttotal | *120 [seconds]* |
| recurs | *2* |
| weight | *1* |
| SB | *4* |
| SN | *5* |
| minint | *1x10^4^* |
| maxint | *1 x10^7^* |
| ended | *1* |
