# Supplementary material for: Nontargeted homologue series extraction from hyphenated high resolution mass spectrometry data
Source: J Cheminform. 2017 Feb 23;9:12. doi: 10.1186/s13321-017-0197-z (PMC5323340; doi:10.1186/s13321-017-0197-z)
Supplement: Supplementary file 6 — Additional file 6. Parameters for series detection. [file 13321_2017_197_MOESM6_ESM.docx]

Table S3. Parameters used for series extraction, R *nontarget* package, function *homol.search()*. See package manual for parameter descriptions.

| **Parameter** | **Article symbol** | **Value** |
| --- | --- | --- |
| elements | *-* | *FALSE* |
| use_C | *-* | *TRUE* |
| minmz | Δ*m/z_min_* | *3* |
| maxmz | Δ*m/z_max_* | *80* |
| minrt | *ΔRT_min_* | *-2 [minutes]* |
| maxrt | *ΔRT_max_* | *2 [minutes]* |
| ppm | *related to ε* | *TRUE* |
| mztol | *ε* | *3* |
| rttol^1^ | *ΔΔRT* | *0.2* |
| minlength | *n_min_* | *5* |
| mzfilter | *-* | *FALSE* |
| spar^2^ | *related to λ* | *0.45* |
| R2 | *R^2^* | *0.98* |

^1^ Not to be confused with the parameter in Table S-5.

^2^ Check documentation of R function *smooth.spline()* for how this parameter is defined.
