# Supplementary material for: Nontargeted homologue series extraction from hyphenated high resolution mass spectrometry data
Source: J Cheminform. 2017 Feb 23;9:12. doi: 10.1186/s13321-017-0197-z (PMC5323340; doi:10.1186/s13321-017-0197-z)
Supplement: Supplementary file 7 — Additional file 7. Blank subtraction parameters. [file 13321_2017_197_MOESM7_ESM.docx]

Table S4. Blank subtraction settings, R *enviMass* package, function *find.raw()*. See package manual for parameter descriptions.

| **Parameter** | **Value** |
| --- | --- |
| dmz | *5* |
| ppm | *TRUE* |
| dRT | *2 [minutes]* |
| int | *0.1 * peak intensity* |
| kdTree | *FALSE* |
