# Supplementary material for: Nontargeted homologue series extraction from hyphenated high resolution mass spectrometry data
Source: J Cheminform. 2017 Feb 23;9:12. doi: 10.1186/s13321-017-0197-z (PMC5323340; doi:10.1186/s13321-017-0197-z)
Supplement: Supplementary file 8 — Additional file 8. Isotopologue grouping parameters. [file 13321_2017_197_MOESM8_ESM.docx]

Table S5. Isotopologue grouping parameters used for the function *pattern.search2()*, *nontarget* R package. See package manual for further parameter descriptions.

| **Parameter** | **Value** |
| --- | --- |
| mztol | *3* |
| ppm | *TRUE* |
| inttol | *0.3* |
| rttol* | *0.1 [minutes]* |
| use_isotopes | *FALSE* |
| use_charges | *FALSE* |
| use_marker | *TRUE* |
| quick | *TRUE* |

* Not to be confused with the parameter in Table S-3.
