# Supplementary material for: Nontargeted homologue series extraction from hyphenated high resolution mass spectrometry data
Source: J Cheminform. 2017 Feb 23;9:12. doi: 10.1186/s13321-017-0197-z (PMC5323340; doi:10.1186/s13321-017-0197-z)
Supplement: Supplementary file 9 — Additional file 9. STP peak and series detection characteristics. [file 13321_2017_197_MOESM9_ESM.docx]

Table S6. Series detection characteristics for the 10 STP effluent samples. The second-last column only counts the series which were paired with at least one other series, whereas the last column states all unique - and possibly multiple per series – pairings below the given intersection angle *θ*. Numbers in brackets state percentages of all observed peaks, series or series pairs.

| **ID** | **Location** | **Ionization mode** | **Peaks** | **Peaks in series** | **Series** | **Series,**  **≠ blank** | **Series, monoisotopic** | **Series, paired** | **Series pairs** ***θ*<0.08π** |
| --- | --- | --- | --- | --- | --- | --- | --- | --- | --- |
| **-** | (Blind sample) | positive  negative | *12843*  *6768* | *1621 (12.6)*  *452 (6.7)* | *573*  *155* | *-*  *-* | *540 (94.2)*  *143 (92.3)* | *514 (89.7)*  *137 (88.4)* | *960 (54.4)*  *263 (85.4)* |
| **1** | Affoltern, Zwillikon | positive  negative | *20788*  *9533* | *7342 (35.5)*  *1099 (11.5)* | *6937*  *822* | *6089 (87.8)*  *749 (91.1)* | *4713 (67.9)*  *714 (86.9)* | *6849 (98.7)*  *792 (96.4)* | *163560 (80.6)*  *23744 (88.4)* |
| **2** | Winterthur | positive  negative | *18024*  *9748* | *5173 (28.7)*  *1054 (10.8)* | *4553*  *1228* | *3777 (83.0)*  *1129 (91.9)* | *2625 (57.7)*  *1174 (95.6)* | *4481 (98.4)*  *1198 (97.6)* | *224561 (93.7)*  *242155 (98.7)* |
| **3** | Werdhölzli, Zürich | positive  negative | *19614*  *10331* | *7135 (36.4)*  *1324 (12.8)* | *6692*  *911* | *6263 (93.6)*  *861 (94.5)* | *4404 (65.8)*  *662 (72.7)* | *6609 (98.8)*  *887 (97.4)* | *122861 (63.1)*  *10469 (72.9)* |
| **4** | Thal, Altenrhein | positive  negative | *21828*  *9721* | *7425 (34.0)*  *1247 (12.8)* | *5641*  *711* | *4947 (87.7)*  *641 (90.2)* | *4013 (71.1)*  *619 (87.1)* | *5532 (98.1)*  *675 (94.9)* | *108869 (69.3)*  *3478 (46.3)* |
| **5** | Uetendorf, Thun | positive  negative | *21105*  *10483* | *7740 (36.7)*  *1236 (11.8)* | *7806*  *715* | *7214 (92.4)*  *670 (93.7)* | *4671 (59.8)*  *606 (84.8)* | *7734 (99.1)*  *688 (96.2)* | *277329 (81.9)*  *5844 (74.2)* |
| **6** | Bioggio, Lugano | positive  negative | *18416*  *10491* | *5909 (32.1)*  *1354 (12.9)* | *4871*  *813* | *4062 (83.4)*  *749 (92.1)* | *3368 (69.1)*  *660 (81.2)* | *4789 (98.3)*  *783 (96.3)* | *57756 (57.3)*  *5707 (56.4)* |
| **7** | Verniere, Aïre | positive  negative | *23336*  *11120* | *8960 (38.4)*  *1419 (12.8)* | *7666*  *741* | *7023 (91.6)*  *692 (93.4)* | *5259 (68.6)*  *617 (83.3)* | *7563 (98.7)*  *692 (93.4)* | *150723 (58.1)*  *3830 (54.0)* |
| **8** | Bussigny-prés-Lausanne | positive  negative | *26409*  *12116* | *14954 (56.6)*  *2401 (19.8)* | *18254*  *2040* | *18118 (99.3)*  *2001 (98.1)* | *12211 (66.9)*  *1595 (78.2)* | *18167 (99.5)*  *1988 (97.5)* | *310782 (31.2)*  *51026 (80.7)* |
| **9** | Hallau, Klettgau | positive  negative | *24958*  *11064* | *11686 (46.8)*  *2019 (18.2)* | *10090*  *1706* | *9694 (96.1)*  *1670 (97.9)* | *6730 (66.7)*  *1331 (78.0)* | *9991 (99.0)*  *1674 (98.1)* | *103496 (37.9)*  *15526 (33.7)* |
| **10** | Schönau, Zug | positive  negative | *17060*  *9582* | *4910 (28.8)*  *864 (9.0)* | *3254*  *488* | *2648 (81.4)*  *440 (90.2)* | *2213 (68.0)*  *373 (76.4)* | *3175 (97.6)*  *460 (94.3)* | *37975 (75.7)*  *5838 (92.2)* |
