# Supplementary material for: Nontargeted homologue series extraction from hyphenated high resolution mass spectrometry data
Source: J Cheminform. 2017 Feb 23;9:12. doi: 10.1186/s13321-017-0197-z (PMC5323340; doi:10.1186/s13321-017-0197-z)
Supplement: Supplementary file 10 — Additional file 10. STP series detection characteristics following randomization. [file 13321_2017_197_MOESM10_ESM.docx]

Table S7. Series detection for the 10 STP effluent samples for the original peaks and false discovery rates after randomization of peak characteristics. For the latter, *RT* values were randomly exchanged among peaks, using the R sample() function without replacement. Randomization was repeated 30 times for each location and ionization mode; values in the two last columns hence state medians and standard deviations. Numbers in brackets state percentages of all observed peaks or series.

| **ID** | **Location** | **Ionization mode** | **Peaks** | **Peaks in series** | **Series** | **Peaks in series, after randomization** | **Series, after randomization** |
| --- | --- | --- | --- | --- | --- | --- | --- |
| **-** | (Blind sample) | positive  negative | *12843*  *6768* | *1621 (12.6)*  *452 (6.7)* | *573*  *155* | *316 (2.5) ± 39*  *22 (0.4) ± 10* | *71 ± 10*  *5 ± 2* |
| **1** | Affoltern, Zwillikon | positive  negative | *20788*  *9533* | *7342 (35.5)*  *1099 (11.5)* | *6937*  *822* | *226 (1.1) ± 29*  *57 (0.5) ± 14* | *48 ± 7*  *13 ± 3* |
| **2** | Winterthur | positive  negative | *18024*  *9748* | *5173 (28.7)*  *1054 (10.8)* | *4553*  *1228* | *231 (1.3) ± 37*  *56 (0.5) ± 17* | *50 ± 8*  *12 ± 4* |
| **3** | Werdhölzli, Zürich | positive  negative | *19614*  *10331* | *7135 (36.4)*  *1324 (12.8)* | *6692*  *911* | *213 (1.1) ± 26*  *65 (0.6) ± 17* | *46 ± 6*  *14 ± 4* |
| **4** | Thal, Altenrhein | positive  negative | *21828*  *9721* | *7425 (34.0)*  *1247 (12.8)* | *5641*  *711* | *246 (1.1) ± 28*  *50 (0.5) ± 16* | *53 ± 7*  *11 ± 4* |
| **5** | Uetendorf, Thun | positive  negative | *21105*  *10483* | *7740 (36.7)*  *1236 (11.8)* | *7806*  *715* | *250 (1.2) ± 39*  *70 (0.6) ± 17* | *53 ± 9*  *15 ± 4* |
| **6** | Bioggio, Lugano | positive  negative | *18416*  *10491* | *5909 (32.1)*  *1354 (12.9)* | *4871*  *813* | *240 (1.3) ± 38*  *76 (0.7) ± 18* | *51 ± 8*  *17 ± 4* |
| **7** | Verniere, Aïre | positive  negative | *23336*  *11120* | *8960 (38.4)*  *1419 (12.8)* | *7666*  *741* | *336 (1.4) ± 37*  *81 (0.7) ± 18* | *72 ± 8*  *17 ± 4* |
| **8** | Bussigny-prés-Lausanne | positive  negative | *26409*  *12116* | *14954 (56.6)*  *2401 (19.8)* | *18254*  *2040* | *1071 (4.1) ± 64*  *91 (0.7) ± 22* | *249 ± 17*  *21 ± 5* |
| **9** | Hallau, Klettgau | positive  negative | *24958*  *11064* | *11686 (46.8)*  *2019 (18.2)* | *10090*  *1706* | *546 (2.2) ± 55*  *100 (0.9) ± 31* | *121±13*  *22 ± 7* |
| **10** | Schönau, Zug | positive  negative | *17060*  *9582* | *4910 (28.8)*  *864 (9.0)* | *3254*  *488* | *208 (1.2) ± 36*  *54 (0.6) ± 18* | *44±8*  *12 ± 4* |
