# Supplementary material for: Nontargeted homologue series extraction from hyphenated high resolution mass spectrometry data
Source: J Cheminform. 2017 Feb 23;9:12. doi: 10.1186/s13321-017-0197-z (PMC5323340; doi:10.1186/s13321-017-0197-z)
Supplement: Supplementary file 11 — Additional file 11. LC-HRMS peaks and series of the SPAC surfactant. [file 13321_2017_197_MOESM11_ESM.docx]

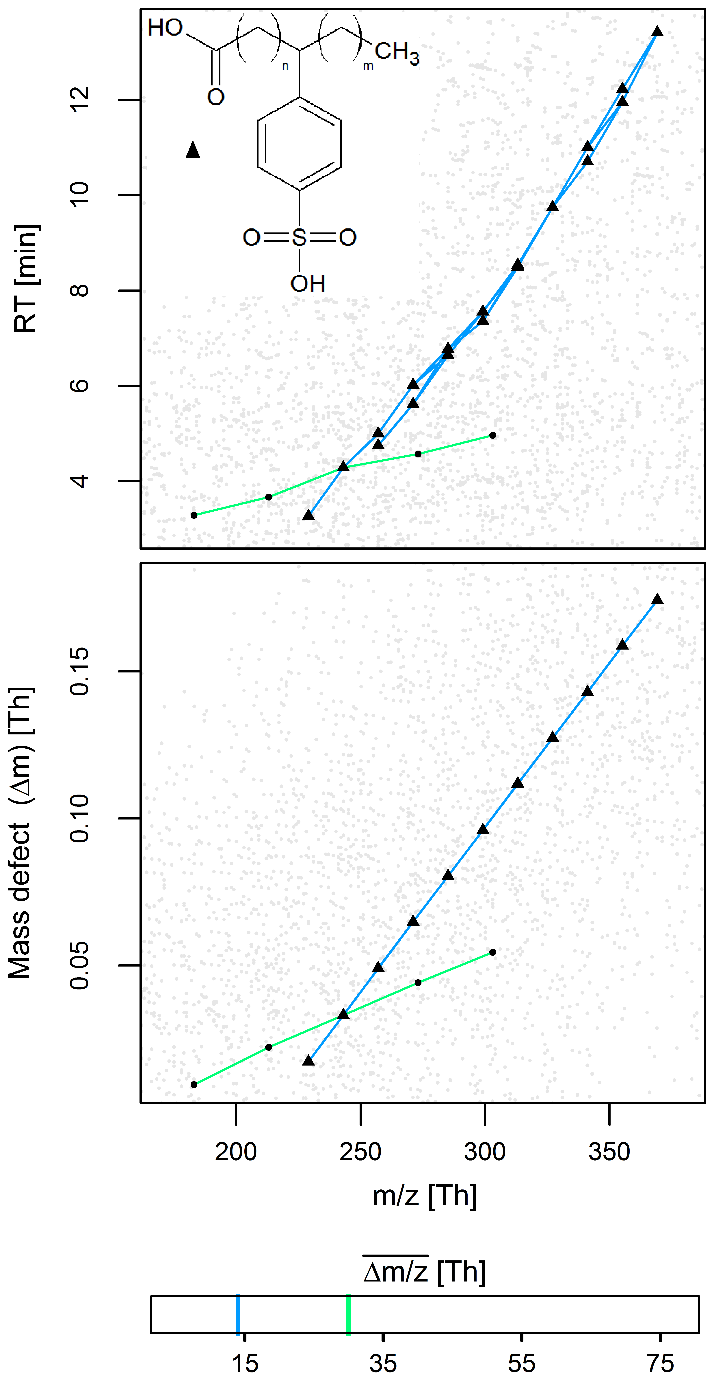


Figure S2. Peaks of 18 partly isobaric homologues of the SPAC surfactant (black triangles, molecular structure in top panel) and the resulting 31 superjacent series detected at $\bar{\Delta m/z}$ ≈ 14.016 *Th* (blue lines). Note that these series and isobaric peaks overlay each other in the lower panel. Another yet unidentified series at $\bar{\Delta m/z}$ ≈ 30.011 *Th* intersects with one SPAC homologue and may indicate the existence of homologue units other than CH_2_ (green line). All other peaks are shown in gray.
