# Supplementary material for: Nontargeted homologue series extraction from hyphenated high resolution mass spectrometry data
Source: J Cheminform. 2017 Feb 23;9:12. doi: 10.1186/s13321-017-0197-z (PMC5323340; doi:10.1186/s13321-017-0197-z)
Supplement: Supplementary file 12 — Additional file 12. Meshed structure of PEG and other yet unidentified LC-HRMS peak series. [file 13321_2017_197_MOESM12_ESM.docx]

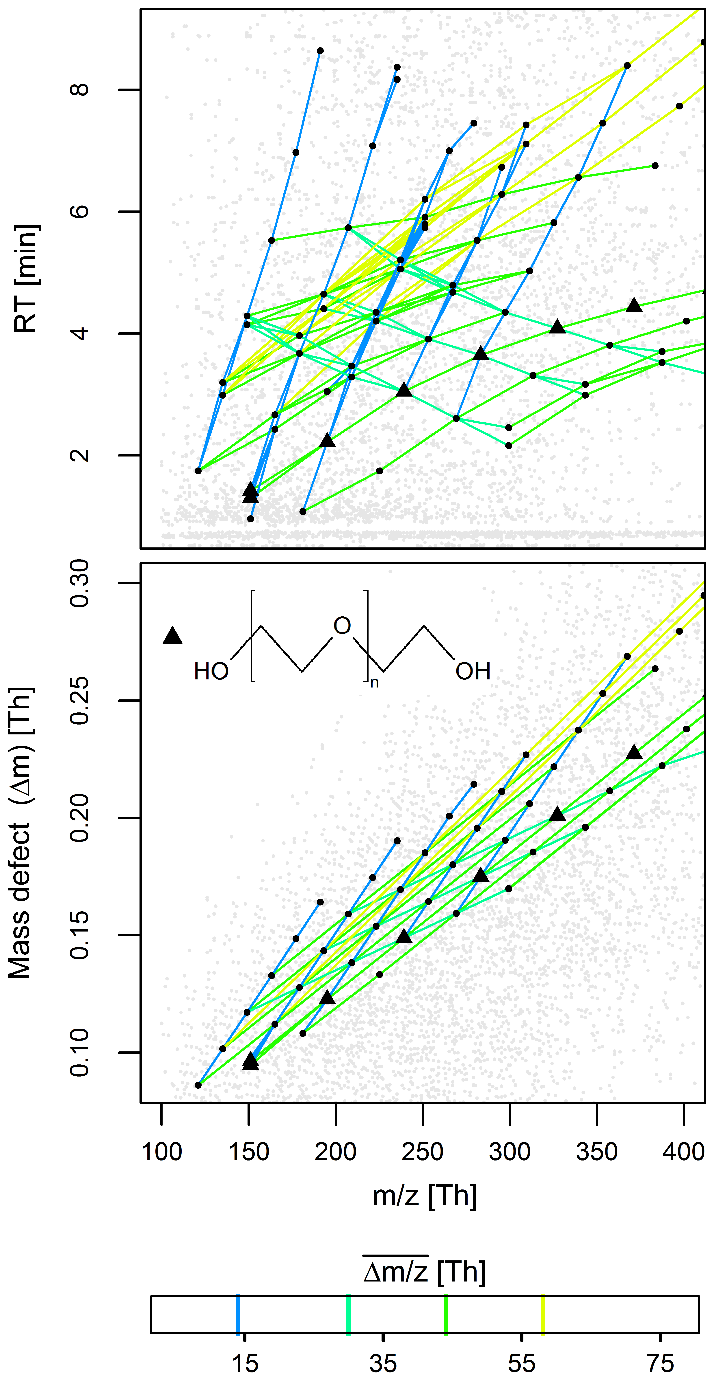


Figure S3. Meshed structure of yet unidentified series peaks (black dots) relatable through mass differences of $\bar{\Delta m/z}$ = 14.016, 30.011, 44.026 and 58.042 *Th* to an identified PEG series (black triangles, molecular structure shown in the lower panel). All other measured peaks are shown in gray. The series were selected from SOM nodes 6-11 of Figure 2 (main text) and Figure S-4 (Additional file 13), e.g., the pairs of series with $\bar{\Delta m/z}$ = 30.011 and 44.026 *Th* map onto SOM node 6 whereas pairs of series with $\bar{\Delta m/z}$ = 14.016 and 58.042 *Th* map onto other SOM regions such as node 4.
