# Supplementary material for: Nontargeted homologue series extraction from hyphenated high resolution mass spectrometry data
Source: J Cheminform. 2017 Feb 23;9:12. doi: 10.1186/s13321-017-0197-z (PMC5323340; doi:10.1186/s13321-017-0197-z)
Supplement: Supplementary file 13 — Additional file 13. SOM results on ΔRT for paired series detected in the Affoltern STP effluent, Switzerland. [file 13321_2017_197_MOESM13_ESM.docx]

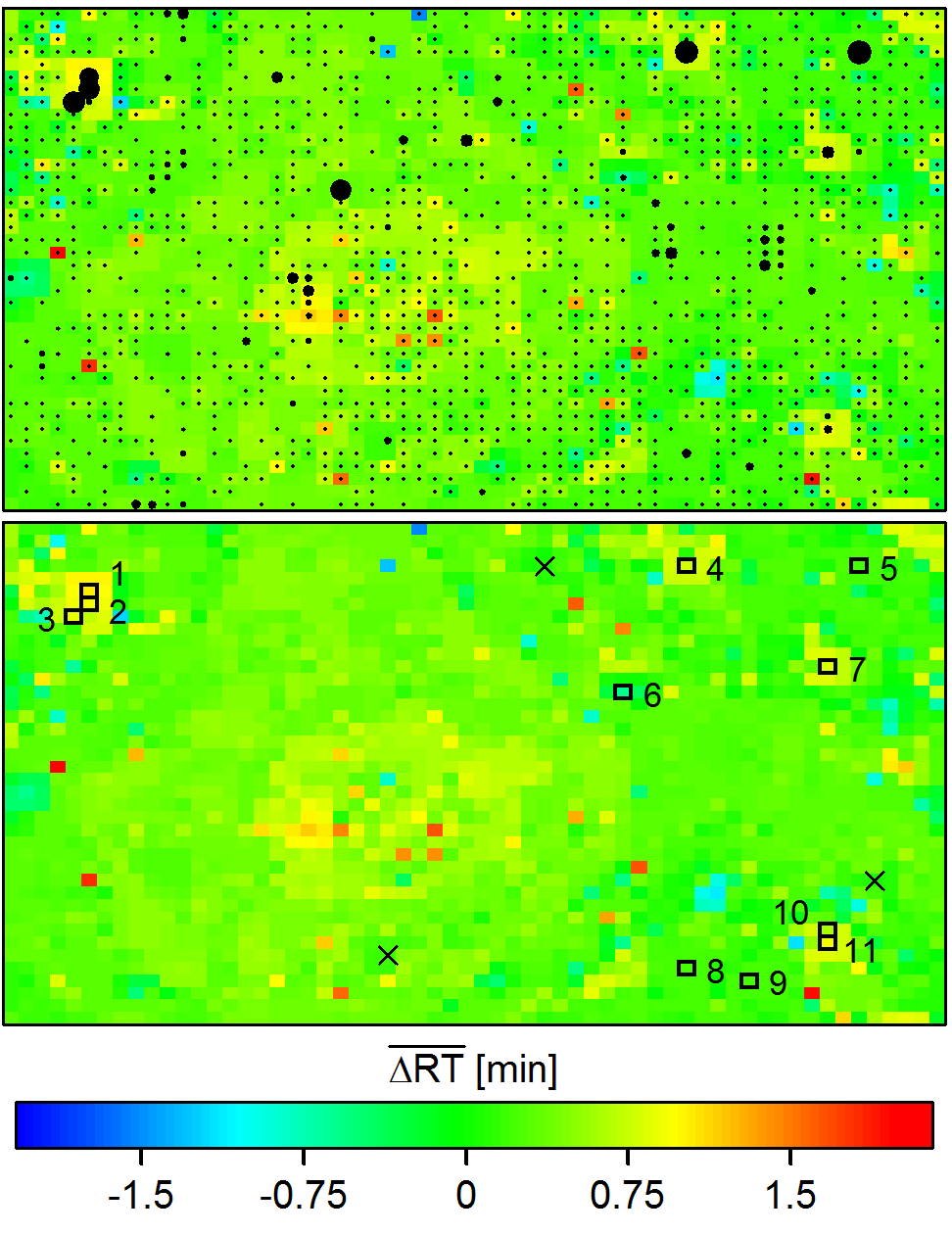


Figure S4. SOM results for all series pairs detected in the STP sample with ID=1 (positive mode), complementing Figure 2 in the main article. Coloring of top and bottom panels depict the $\bar{\Delta{RT}_{x}}$ and $\bar{\Delta{RT}_{y}}$ values at the SOM grid nodes, respectively. Sizes of the black dots in the top panel indicate frequencies of monoisotopic series pairs mapped onto the nodes. In turn, black squares in the bottom panel either indicate the three nodes with highest frequencies at low intersection angles (*θ<0.08π, nodes 1-3*) or nodes with highest mapping frequencies containing 50% of all monoisotopic series pairs at large intersection angles *(θ≥0.08π, nodes 4-11)*. The series mapped onto the latter nodes are shown in Figure 3. Moreover, crosses highlight the mapping nodes of the superjacent series shown in Figure S-6 (Additional File 15).
