# Supplementary material for: Nontargeted homologue series extraction from hyphenated high resolution mass spectrometry data
Source: J Cheminform. 2017 Feb 23;9:12. doi: 10.1186/s13321-017-0197-z (PMC5323340; doi:10.1186/s13321-017-0197-z)
Supplement: Supplementary file 14 — Additional file 14. Histogram of intersection angles between paired SOM series. [file 13321_2017_197_MOESM14_ESM.docx]

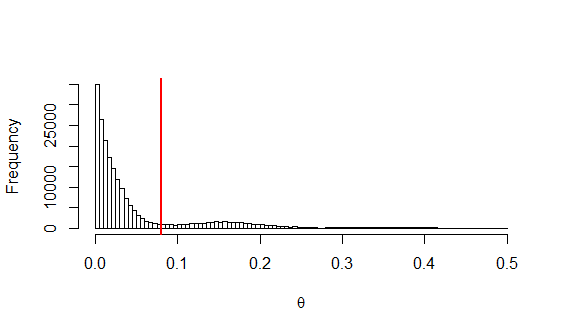


Figure S5. Distribution of intersection angle *θ* in series pairs of STP sample with ID=1. The red line signifies a threshold of θ*=0.08π* (equivalent to *14.4°*), selected from a local minimum gap between the two modes at θ*=0π* and θ*=0.15π.*
