# Supplementary material for: Nontargeted homologue series extraction from hyphenated high resolution mass spectrometry data
Source: J Cheminform. 2017 Feb 23;9:12. doi: 10.1186/s13321-017-0197-z (PMC5323340; doi:10.1186/s13321-017-0197-z)
Supplement: Supplementary file 15 — Additional file 15. Superjacent series exemplification. [file 13321_2017_197_MOESM15_ESM.docx]

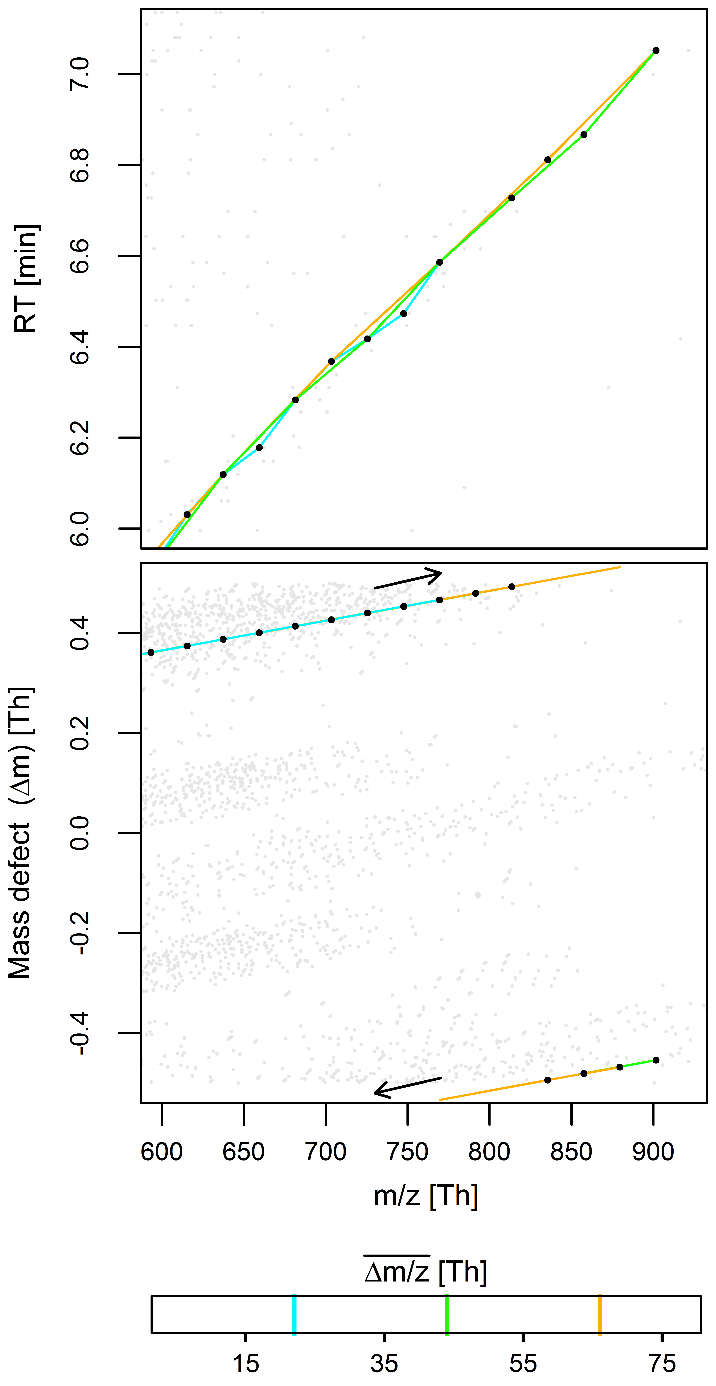


Figure S6. Example of 3 superjacent series for STP sample ID=1, positive ionization. Blue, green and orange lines connect series peaks at $\bar{\Delta m/z}$ ≈ 22.013, 44.026 and 66.039 [Th], respectively. Peaks not part of these series are shown as gray instead of black dots. The three nodes onto which the series pairs are mapped are highlighted as crosses in Figures 2 (main text) as well as Figures S-4 and S-7 (Additional files 13 and 16).
