# Supplementary material for: Nontargeted homologue series extraction from hyphenated high resolution mass spectrometry data
Source: J Cheminform. 2017 Feb 23;9:12. doi: 10.1186/s13321-017-0197-z (PMC5323340; doi:10.1186/s13321-017-0197-z)
Supplement: Supplementary file 16 — Additional file 16. Intersection angle θ of SOM nodes. [file 13321_2017_197_MOESM16_ESM.docx]

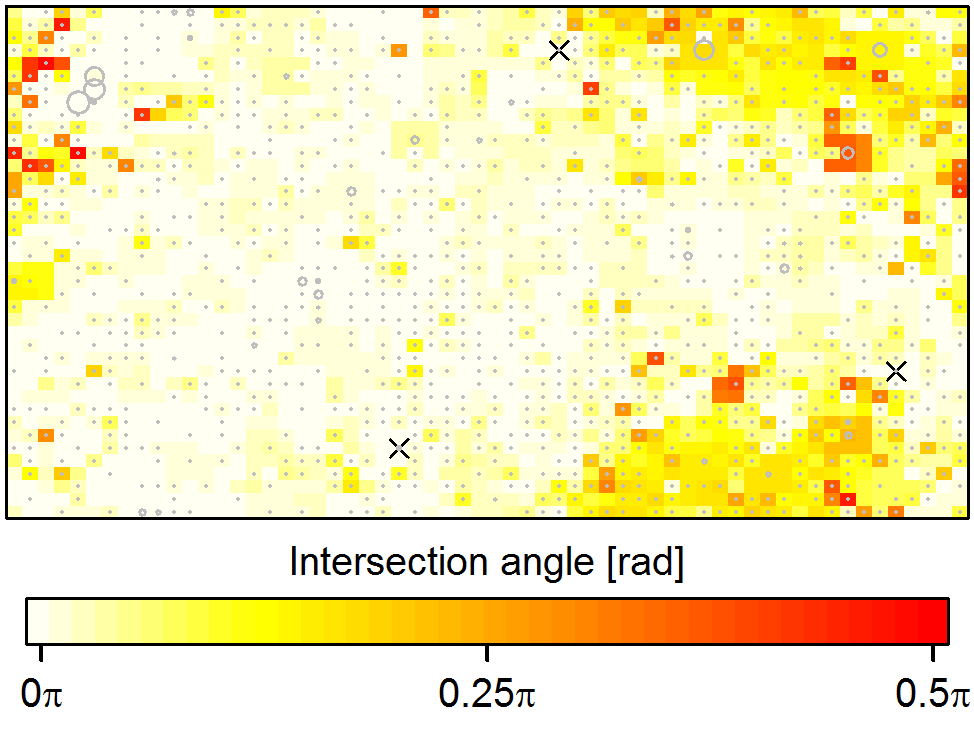


Figure S7. Distribution of intersection angle θ at the SOM nodes trained for the series pairs detected in the STP sample with ID=1 (positive mode). Sizes of the gray circles indicate frequencies of monoisotopic series pairs mapped onto individual SOM nodes. Crosses highlight the mapping nodes of the superjacent series shown in Figure S-6 (Additional File 15).
